# Polysonographic changes in obese patients with indication of bariatric surgery

**DOI:** 10.1590/0100-6991e-20213030

**Published:** 2021-11-05

**Authors:** VICTOR MARTINS FERNANDES, GIBRAN RIBEIRO DA ROCHA, THIAGO CARVALHO MILET, DANIEL MATOS BARRETO, JORGE FARIA DE MIRANDA SANTOS, MONICA MEDRADO OLIVEIRA

**Affiliations:** 1 - Universidade Estadual do Sudoeste da Bahia, Departamento de Ciências Naturais, Faculdade de Medicina - Vitória da Conquista - BA - Brasil; 2 - Hospital Geral de Vitória da Conquista, Cirurgia Geral - Vitória da Conquista - BA - Brasil; 3 - Cirurgia do Aparelho Digestivo e Obesidade, Salvador - BA - Brasil; 4 - Centro Especializado em Pneumologia e Distúrbios do Sono, Salvador - BA - Brasil; 5 - Núcleo de Tratamento e Cirurgia da Obesidade, Salvador - BA - Brasil

**Keywords:** Polysomnography, Bariatric Surgery, Obesity, Sleep Apnea, Obstructive, Polissonografia, Cirurgia Bariátrica, Obesidade, Síndromes da Apneia do Sono

## Abstract

**Introduction:**

obstructive Sleep Apnea Syndrome (OSAS) is a serious confition that compromises the quality of life and survival of patients. Its main risk fator in adults is obesity and the gold standard test for diagnosis is polysomnography (PSG), mainly through the apneia-hypopnea index (AHI)*.* **Objective:** to analyze the sleep pattern of obese patients with indication for bariatric surgery, determining the main polisomnographic parameters compromised by obesity.

**Methods:**

This work is a cross-sectional study with analysis of polysomnography perfomed in patients with obesity in the peroperative period of bariatric surgery at a clinic in Vitória da Conquista/BA during 2017. The Epi Info 7 platform was used for analysis of the data*.*

**Results:**

58 polysomnographic reports were analyzed, with 56,9% morbdly obese and 43,1% non-morbid. The prevalence of OSAS was 70,68% and de AHI ranged from zero to 84,6 with a mean of 19,47±22,89 e/h. morbidly obese, compared to “non-morbid”, had a longer saturation time below 80% and 90% (0,4±0,93 vs. 0,12±0,45 e 4,87±7,38 vs. 1,36±2,87 respectively; p-value=0,02 in both), worse index respiratory disorders ((29,24±25,36 vs. 16,88±16,21; p-value=0,02), higher AHI (24,71±25,68 vs. 12,56±16,67; p-value=0,02), higher hypopnea index values (16,41±17,10 vs. 6,99±8,52; p-value=0,006) and lower minimum saturation (78,24±9,80 vs. 85,24±6,33; p-value=0,004)*.*

**Conclusions:**

the high prevalence of OSAS found confirms its indication in the preoperative period of bariatric surgery. The main respiratory event involved in most individuals with OSAS was the hypopnea index.

## RESUMO

**Introdução:**

a Síndrome da Apnéia e Hipopnéia Obstrutiva do Sono (SAHOS) é uma grave patologia que compromete a qualidade de vida e sobrevida dos portadores. Seu principal fator de risco em adultos é a obesidade e o exame padrão ouro para diagnóstico é a polissonografia (PSG), sobretudo através do índice de apneiahipopneia (IAH).

**Objetivo:**

analisar o padrão de sono dos pacientes obesos com indicação à cirurgia bariátrica, determinando os principais parâmetros polissonográficos comprometidos pela obesidade.

**Métodos:**

Este trabalho se trata de um estudo transversal com análise das polissonografias feitas em pacientes com obesidade no préoperatório de cirurgia bariátrica em uma clínica em Vitória da Conquista/BA durante o ano de 2017. Foi utilizada a plataforma Epi Info 7 para análise dos dados.

**Resultados:**

analisados 58 laudos polissonográficos, sendo 56,9% obesos mórbidos e 43,1% não mórbidos. A prevalência de SAHOS foi 70,68% e o IAH variou de zero a 84,6 com média de 19,47±22,89 e/h. Os obesos mórbidos, comparados aos “não mórbidos”, tiveram maior tempo de saturação abaixo de 80% e 90% (0,4±0,93 vs. 0,12±0,45 e 4,87±7,38 vs. 1,36±2,87 respectivamente; p=0,02 em ambos), pior índice de distúrbios respiratórios (29,24±25,36 vs. 16,88±16,21; p=0,02), maior IAH (24,71±25,68 vs. 12,56±16,67; p=0,02), valores de índices de hipopneia superiores (16,41±17,10 vs. 6,99±8,52; p=0,006) e menor saturação mínima (78,24±9,80 vs. 85,24±6,33; p=0,004).

**Conclusões:**

a alta prevalência de SAHOS encontrada ratifica sua indicação no pré-operatório de cirurgia bariátrica. O principal evento respiratório envolvido na maioria dos indivíduos com SAHOS foi o índice de hipopneia.

## INTRODUCTION

The Obstructive Sleep Apnea and Hypopnea Syndrome (OSAHS) is a sleep-related respiratory disorder characterized by upper airway obstruction that results in a cycle of hypoxemia, increased work of breathing, and frequent micro-arousals^1^. As obesity represents a very prevalent and reversible risk factor for OSAHS in adults^2^, its global increase directly impacts the syndrome rates^3^. There is a predominance in obese and middle-aged individuals, reaching more than 40%, substantially higher than the prevalences of 2% in women and 4% in men in the general population^4^ ^,^ ^5^. Along with systemic arterial hypertension (SAH), OSAHS is one of the most prevalent comorbidities of obese patients in the preoperative period of bariatric surgery^6^ ^,^ ^7^. 

Although the pathophysiological mechanism is not yet fully elucidated, it is accepted that the adipose tissue in the neck compresses and narrows the upper airway lumen, inducing it to collapse, causing a drop in oxyhemoglobin saturation, an increase in adrenergic discharge, and its consequent clinical manifestations, which are better analyzed through polysomnography^8^ ^-^ ^10^.

OSAHS is related to several morbidities, such as SAH, acute myocardial infarction, stroke, traffic accidents, among others, and is therefore considered a public health problem^11^. It chronically alters the sensitivity of the peripheral chemoreflex, causes cardiovascular dysfunction and metabolic dysregulation associated with varied symptoms, mainly daytime sleepiness, nocturnal awakening, and nocturnal suffocation, especially in the long term^12^ ^,^ ^13^. Even so, most patients with the syndrome are asymptomatic, which does not mean a milder disease^13^.

The standard exam for OSAHS diagnosis is type 1 polysomnography (PSG) and currently most bariatric surgery programs have PSG as a routine preoperative assessment^14^. In general, the syndrome reaches its incidence peak around the sixth decade of life, but in individuals with a relevant BMI increase, this peak moves to the fifth decade^2^. The objective of this work is to identify the polysomnographic alterations found in obese patients in the preoperative period of bariatric surgery.

## METHOD

This is a cross-sectional study with analysis of polysomnographies performed in obese patients in a clinic located in Vitória da Conquista, state of Bahia (BA), Brazil. 

We selected all 58 patients who underwent the examination in a sleep medicine facility in the preoperative evaluation of bariatric surgery during 2017, in Vitória da Conquista/BA. We analyzed the results using the Epi Info 7 software.

The research followed the recommendations of the American Academy of Sleep Medicine (AASM) regarding polysomnographic criteria, which classify OSAHS taking into account the apnea/hypopnea index (AHI), which reflects the number of apneas and hypopneas per hour of sleep. The condition is considered mild when the AHI is between 5 and 15, moderate when between 15 and 30, and severe when greater than 30. Values below 5 are considered normal.

The variables studied were age, sex, weight, body mass index (BMI), height, total sleep time (TST), sleep latency (LATENCY), REM sleep latency (REMLAT), sleep efficacy (SE), non-REM sleep time 1 (NREM1), non-REM sleep time 2 (NREM2), non-REM sleep time 3 (NREM3), REM sleep time (REM), time wake after sleep onset (WASO), Arousals Index (AROUI), Periodic Limb Movements Index (PLM), Respiratory Disturbances Index (RDI), Apnea-Hypopnea Index (AHI), Apnea Index (AI), Hypoapnea Index (HI), average saturation (AVSAT), minimum saturation (MINSAT), time with blood oxygen saturation below 90% (SAT <90%) and time with blood oxygen saturation below 80% (SAT <80%).

We divided patients according to BMI in morbidly obese (grade 3) and non-morbidly obese (grades 2 and 1). As for age, we considered two groups, one with patients aged 39 years or less and another containing patients aged 40 years or more. Finally, regarding AHI, we divided the patients twice, both into two groups: the first had groups of patients classified as “normal and mild” versus “moderate and severe”, and the other considered “non-apneic” versus “apneic” patients. It is noteworthy that, during the development of the study, we considered patients with OSAHS based only on polysomnographic parameters, since we did not analyze clinical complaints. 

The research project was evaluated and approved by the Ethics in Research Committee (CEP) through Plataforma Brasil under the Ethical Appreciation Presentation Registry (CAAE) number 09397619.4.0000.0055.

## RESULTS

Were conducted and studied 58 polysomnography exams, 56.9% (33) in degree 3 obese individuals and 43.1% (25) in non-morbidly ones, the latter group consisting of 36.2% (21) obesity grade 2 and 6.9% (4) grade 1. Most of the sample consisted of females, equivalent to 84.48% (49), men representing 15.52% (9). Age ranged from 21 to 61 years, with a mean of 38.17± .54. BMI ranged from 33.30 to 66.90 kg/m², with a mean of 41.37±5.83. AHI ranged from zero to 84.6 events per hour, with a mean of 19.47±22.89. MINSAT ranged from 59% to 94%, with a mean of 81.25±9.11%, and AVSAT ranged from 90% to 98%, with a mean of 94.86±1.73%. The minimum, maximum, and average values of SAT <80% were 0%, 4.7%, and 0.28%±0.77%, and of SAT <90% were 0%, 31.8%, and 3.36±6.09%, respectively (Table 1). 

**Table 1:** Minimum, maximum, and average of RDI, AHI, AI, HI, LATENCY, REMLAT, NREM1, NREM2, NREM3, REM, AVSAT, MINSAT, AROUI, SE, TST, WASO, weight, height and BMI of obese patients in preoperative evaluation of bariatric surgery.

	Average	SD	Maximum	Minimum
RDI (events/hour)	23,91	22,58	86	2
AHI (events/hour)	19,47	22,89	84,60	0,20
AI				
(events/hour)	7,03	12,58	60,10	0
HI (events/hour)	12,35	14,72	67,40	0,20
LATENCY (minutes)	13,78	22,20	160,40	0
REMLAT (minutes)	109,94	50,28	298,50	49
NREM1	8,69%	5,77	28%	1,40%
NREM2	47,90%	8,63	68%	22,80%
NREM3	21,99%	9,15	53,40%	6%
REM	21,52%	4,41	32%	10,20%
AVSAT	94,86%	1,73	98%	90%
MINSAT	81,25%	9,11	94%	59%
SAT<80%	0,28%	0,77	4,7%	0%
SAT<90%	3,36%	6,09	31,8%	0%
AROUI (events/hour)	21,91	14,98	74,40	5,90
SE	86,21%	8,92	97,30%	50,20%
TST (minutes)	403	51,76	489	223,50
WASO (minutes)	41,98	28,32	143	5,50
Weight (kg)	110,90	17,59	191	81
Height (m)	1,63	0,0826	1,88	1,48
BMI (kg/m²)	41,37	5,83	66,90	33,30
Age (years)	38,17	9,54	61	21

 Of the total number of patients, 56.9% (33) were under 40 years of age and 43.1% (25) in the 40 years or older group. As for AHI, 63.79% (37) were classified as “normal and mild” and 36.21% (21) belonged to the “moderate and severe” group. Only 29.32% (17) patients were classified as “non-apneic”.

The group of “morbidly obese” showed worsening of polysomnographic parameters when compared to the group of “non-morbidly obese” in the SAT <80% (p=0.02), SAT <90% (p=0.02), RDI (p=0.02), HI (p=0.006), AHI (p=0.02), and MINSAT (p=0.004), as shown in Table 2. 

**Table 2:** Relation of the variables SAT<80%, SAT<90%, RDI, AHI, HI and MINSAT in the diferente groups of obese individuals.

	Obeses	Average	SD	X²	p-value
SAT<80%	Morbidly obese	0.4%	0.93	4.81	
	Non-morbidly obese	0.12%	0.45	0.02*	
SAT<90%	Morbidly obese	4.87%	7.38	4.8	
	Non-morbidly obese	1.36%	2.87	0.02*	
RDI (eventos/hora)	Morbidly obese	29.24	25.36	5.04	
	Non-morbidly obese	16.88	16.21	0.02*	
AHI (eventos/hora)	Morbidly obese	24.71	25.68	5.29	
	Non-morbidly obese	12.56	16.67	0.02*	
HI (eventos/hora)	Morbidly obese	16.41	17.10	7.42	
	Non-morbidly obese	6.99	8.52		0.006*
MINSAT	Morbidly obese	78.24%	9.80	8.28	
	Non-morbidly obese	85.24%	6.33		0.004*

*Mann-Whitney/Wilcoxon Two-Sample Test.

The group of patients with “40 years or more” displayed worse values than the group “under 40 years” regarding AROUI (p=0.002), RDI (p=0.002), AHI (p=0.006), and HI (p=0.01) (Table 3). 

**Table 3:** Analysis of the variables AROUI, RDI, AHI and HI according to age group.

	Age group	Average	SD	X²	p-value
AROUI (events/hour)	40 years or more	28.36/h	18.17	9.08	0.002*
	39 years or less	17.03/h	9.74		
IDRDIR (events/hour)	40 years or more	34.20/h	26.45	9.23	0.002*
	39 years or less	16.11/h	15.43		
AHI (events/hour)	40 years or more	29.46/h	27.57	7.29	0.006*
	39 years or less	11.90/h	15.04		
HI (events/hour)	40 years or more	17.78/h	18.83	5.81	0.01*
	39 years or less	8.24/h	8.94		

*Mann-Whitney/Wilcoxon Two-Sample Test.

 As for the AHI divided in “Moderate and severe” versus “Normal and mild” with other polysomnographic variables, we observed a worse apnea-hypopnea index in patients with higher weight (p=0.007) and BMI (p=0.03), in addition to being related to worse MINSAT (p<0.00001) and AVSAT (p<0.00001), as shown in Table 4.

**Table 4:** Ratio of AHI with weight, BMI, SATMIN and SATMED.

	AHI	Average	SD	p-value
Weight	Moderate and severe	118.90kg	22.22	0.007*
	Normal and mild	106.36kg	12.54	
BMI	Moderate and severe	43.46kg/m²	7.37	0.03**
	Normal and mild	40.18kg/m²	4.43	
MINSAT	Moderate and severe	72.61%	7.38	<0.00001*
	Normal and mild	86.16%	5.75	
AVSAT	Moderate and severe	93.52%	1.44	<0.00001*
	Normal and mild	95.62%	1.40	

*ANOVA **Mann-Whitney/Wilcoxon Two-Sample Test.

When analyzing “apneic” versus “non-apneic” individuals, the AHI showed statistical significance with sex and age group. While females corresponded to 17 “non-apneic” patients and 32 “apneic”, the male group had all the subjects classified as “apneic” (p=0.03). Among individuals under 40 years of age, 20 were in the “apneic” group and 13 in the “non-apneic” one. In patients aged 40 years or more, the values were 21 and four, respectively (p=0.04) (Table 5). 

**Table 5:** Normal patients versus those affected by OSAHS according to gender and age group.

Osahs				
	Non-apneics	Apneics	X²	p-value
39 years or less (n=33)	13	20		
40 years or more (n=25)			2.71	0.04
	4	21		
Total (n=58)	17	41		
Feminine (n=49)	17	32		
Masculine (n=9)	0	9	2.90	0.03*
Total (n=58)	17	41		

*Fisher

The occurrence of sleep apnea was influenced by age, so that the “non-apneic” group had a mean age of 32.29±7.17 years versus 40.60±9.39 years in the “apneic” group (p=0.001). Finally, analyzing the minimum saturation for the “apneic” and “non-apneic” groups was 77.46%±8.09 and 90.41%±2.55, and the mean was 94.2±1.6 and 96.47±0.62, respectively (p<0.00001).

## DISCUSSION

Grade 3 obese patients had more severe AHI values than the ones with grades 1 and 2, showing a direct relationship between the increase in BMI and AHI worsening. When comparing these values with the prevalence of this syndrome in the normal population, an alarming increase can be seen^4^. The study by Tangerina et al.^15^ diverged from the results herein presented in that the average ages of patients affected by OSAHS vs. the non-affected ones were 44.6±10.2 and 53.1±9.1 years, respectively. However, the authors of the study believe that, among the variables involved in OSAHS, age is the least influential if the average is within the values considered as middle age (between 40 and 55 years). 

The proportions found regarding AHI agreed with the findings of Weingarten et al.^16^, who classified 22.45% as non-apneic, 30.99% with mild apnea, 15.93% moderately apneic, and 30.61% affected by severe apnea. Aguiar et al.^17^, on their turn, showed very similar values in all groups: 23.68% non-apneic patients, 26.31% with mild AHI, 23.68% moderately affected, and 26.31% with severe apnea. Finally, of the apneic patients studied by Tangerina et al.^15^, 34.3% had mild disease, 25.7% moderate, and 40% severe (Table 6).

**Table 6:** comparison of the distribution of apnea degrees between the sample of each study.

	Weingarten et al., 2011	Aguiar et al., 2012	Tangerina et al., 2008	Present study
Non-apneic	22.45%	23.68%	^-^	29.31%
Mild apnea	30.99%	26.31%	34.3%	34.48%
Moderate apnea	15.93%	23.68%	25.7%	13.79%
Severe apnea	30.61%	26.31%	40%	22.41%

 Morbidly obese individuals had worse values in the blood oxygen saturation time <80% and <90%, RDI, AHI, HI, and MINSAT, when compared with non-morbidly obese ones. This indicates that the continuous increase in BMI directly interferes with these variables, even in individuals classified as obese. Nevertheless, we could not say the same for the variables SE, NREM1, NREM3, REM and WASO. This is possibly because BMI may have greater relevance when considering the normal weight population versus the obese, especially when separating the eutrophic individuals from the overweight patients, comparing them to obese ones without separating them by degrees. Other authors, such as Tangerina, et al.^15^, also failed to prove the impact of obesity on these variables. 

We observed that middle-aged patients have worse indexes of AROUI, RDI, AHI and HI regardless of obesity, since there was no statistically significant relationship between obesity degrees and age group. Sleep quality is multifactorial, and obesity is not necessarily the main factor involved. However, when present, obesity can worsen and hinder the treatment of several other diseases that share the same risk factors and clinical characteristics, such as metabolic syndrome and atrial fibrillation^18^. 

The relationship between OSAHS and obesity was mainly observed through the increase in hypopneas to the detriment of other variations of sleep-related respiratory disorders, being the main variable found. 

The studied sample differed from the epidemiology described in the literature regarding sex, as it is a procedure sought mainly by the female population, in up to 70%^19^. AHI was directly related to weight and not just BMI, which can be explained by most of the population studied being female and also having higher rates of obesity. 

The discrepancy between the number of patients according to sex may have affected the results and, thus, disagrees with the literature. This effect may have been increased by the population of men studied, all of whom have some degree of OSAHS, and a greater proportion is over 40 years of age. Men sought surgery with a later and worse OSAHS condition, since the search for medical service tends to be delayed, generally when the weight interferes significantly with daily activities^20^. Furthermore, women are also motivated by individual aesthetic issues and even by external interference imposed by the society they belong to, leading them to seek surgical intervention earlier^21^ ^,^ ^22^. Even so, a very similar proportion between sexes was observed by Modena et al.^2^ in the preoperative period of bariatric surgery, confirming that the population profile in surgery clinics may differ from the general population. 

The relationship of OSAHS with age group and sex allows to infer that when the average age is lower or very close to the beginning of the middle age, BMI can be an important and relevant criterion for the development of the syndrome. This association with male sex has been described in other studies and may be due to the distribution of the adipose tissue of obese men, more common in the vicinity of the upper airways, causing worsening of ventilatory mechanics^5^ ^,^ ^23^ ^-^ ^25^.

Classically, OSAHS can be treated with continuous positive airway pressure (CPAP), although with low adherence^4^. Researches have evaluated the impact of CPAP and respiratory physiotherapy on pulmonary mechanics in the pre- and postoperative period of bariatric surgery, with positive results^26^ ^,^ ^27^, which can therefore serve as an adjuvant in the treatment of the syndrome.

Some limitations can be reported in the present study, such as the discrepancy in the number of patients between sexes, the lack of association between the OSAHS degree and the difficulty of clinical control of comorbidities, and, most importantly, the non-performance of control PSG after weight reduction to compare it with the previous result. Studies with a larger number of patients and based on sample calculations can overcome some of the imperfections and be more reliable.

## CONCLUSION

There is a high prevalence of OSAHS and polysomnographic variables associated with obese individuals, which reaffirms the need for screening for the syndrome in this population and can be used to develop specific complementary therapeutic strategies.

Continuous weight gain can further compromise the quality of life and sleep, including the worsening of respiratory disorders, which can interfere with other clinical conditions, worsening prognosis, morbidity, and mortality. The main respiratory event involved in most individuals with OSAHS was the hypopnea index.
